# A Contribution to the Pathology and Cure of Bronchocele

**Published:** 1833-10

**Authors:** 


*Ueber den Kropf. Ein Beitrag zur Pathologie und Therapie desselben*, von Karl Joseph Beck, der Medicin und Chirurgie Doctor, &c. Freiburg, 1833.

*A Contribution to the Pathology and Cure of Bronchocele.* By Dr. Beck. 8vo. pp. 81.

Dr. Beck commences his essay by expressing his dissent from the opinion of Muihlibach, who has ventured to assert that the surgical knife can rarely afford relief in cases of bronchocele. On the contrary, says our author, cases which do not admit of an operation form but a slender minority: among these exceptions are those bronchoceles which are intimately connected with the subjacent parts, or which consist chiefly of a solid substance, and involve the greatest part of the thyroid gland. The superior thyroid artery has been successfully tied by Walther and others in cases of bronchocele caused by primary enlargement of vessels; Hedenus, Gräfe, and Mandt, have extirpated large bronchoceles; and the successful employment of the seton has been revived by Quadri: all showing that, in the very cases in which medicines are useless, and the system is in the highest degree disturbed by the disease, surgical treatment can afford the most perfect relief. "I can myself," says our author, "give a voucher from my own experience for the favourable result of tying the superior thyroid artery, which I had an opportunity of witnessing last summer, in my clinical practice. Practical communications concerning this operation," continues Dr. Beck, "are still to be desired, as the number of persons on whom it is known to have been performed is still too small to give a satisfactory result in an empirical manner, by combining the cases.

"Case. M. W., a woman, aged twenty-two, had suffered several years from a *struma* covering chiefly the right side of the neck. The tumour caused many disagreeable symptoms, which were heightened by its gradual increase. A number of remedies, which are of efficacy against goître, were administered, but without advantage. The swelling, which was warm to the touch, was firm, tense, and somewhat uneven in its lower part. The vascular condition of its superficies could be seen and felt. That the arterial branches entering the tumour were enlarged could be ascertained from the pulsation, which was felt almost throughout it, and from the troublesome throbbing in the tumour of which the patient herself was sensible. She suffered from confusion of the head, dyspnœa, and dysphagia; and, in addition to these local disorders, there was great and general irritability of the vascular system, and inclination to a state of congestion. Yet, with the exception of the enlargement of the superior thyroid artery, and its branches, no disorganization of the vascular system could be perceived. These appearances demonstrated the existence of a *struma aneurysmatica;* nor could we help observing that a change had taken place in the lower cells of the gland, by their being enlarged, and filled with a diseased secretion.

"After the administration of some cooling evacuants, the superior thyroid artery was tied in the clinical ward, on the 2d of July, 1832. An incision was made through the skin and the platysma myoides, at the inner edge of the sternocleido-mastoideus, between the os hyoides and the thyroid cartilage. The artery, which was much enlarged, was then isolated, and tied with a simple loop. I may remark, that I was obliged to enlarge the incision at its upper angle, as a branch took its origin from the artery at the exact spot where I had begun to isolate it. Professor Schwörer had the kindness to assist me in the operation. As soon as the ligature had been fixed, the part of the artery in the direction of the gland sank down, the pulsation at this part ceased, nor could any be felt in the tumour, or in the numerous branches passing over its superficies. The tumour lost its turgescence and was somewhat diminished in circumference, immediately after the operation, as was ascertained by measuring it. The edges of the wound were brought together, and the tumour was covered with ice. The application of cold was continued, even after the ligature came away, which happened on the ninth day. The treatment was antiphlogistic, and the patient was kept quiet, with her head turned towards the affected side. On the second day an inflammatory fever set in, with a coated tongue and increase of the difficulty of swallowing and breathing; but, although the general circulation was much more tumultuous, the extended pulsation of the bronchocele did not recur. On the sixth day after the operation, the uneasy feelings of the patient began to diminish.

"I have merely to remark, in conclusion, that in the third week after the operation, a considerable diminution of the tumour could be perceived, especially in the upper part. In six weeks the tumour was reduced to a third of its previous size, the chief decrease being in its upper part, and less alteration in proportion having taken place in the more indurated part below; yet even here the circumference of the neck was an inch less. The tumour had entirely lost its tension, and was even flaccid. The unpleasant symptoms, of which the patient had formerly complained, no longer existed; but Æthiops antimonialis and burnt sponge were exhibited on account of the strumous spot at the lower part of the gland."

This was a case, observes Dr. Beck, of a *Struma aneurysmatica*. A diseased enlargement existed of the smallest arterial twigs, as well as of the greater branches. This morbid enlargement was of the primitive kind, was connected with a more violent circulation of the general mass of blood, and was originally caused by the flux of blood towards these parts which takes place at the approach of puberty. It cannot be denied that, in consequence of the increased quantity of blood contained in the thyroid gland, the process of nutrition had become disordered, and therefore a tumour still remained, though of trifling size when compared to the former one. This *Struma aneurysmatica* could not be mistaken for a *Struma vasculosa secundaria*, a state which often exists in old bronchoceles of the lymphatic kind, where the enlargement of the vessels is not the cause, but the effect, of the disease. The manner in which it had arisen, as well as its sensible qualities, made the diagnosis easy and certain.

Still, continues Dr. Beck, if we wish to make a proper estimate of this operation in cases of *Struma aneurysmatica*, we must first consider the dangerous condition of the patient for some time after the operation, and then how far it is capable of effecting a cure. Fatal secondary hemorrhages have been frequent in proportion to the small number of cases in which the operation has been performed, which must proceed from some especial cause. In Langenbeck's case, indeed, the hemorrhage obviously depended on the unusual origin and course of the artery tied; for there was no external carotid, but the superior thyroid artery (which was curved) gave off the branches which generally arise from the external carotid. The ligature lay between the place where these branches were given off and the gland. The branches which were given off from the artery, close upon the ligature, prevented the cicatrix holding, when the ligature came away. Dr. Beck's colleague, Buchegger, has several times observed a similar irregularity, namely, that the lingual artery is given off by the superior thyroid, near its origin.

After considering the tendency to secondary hemorrhage caused by these and similar circumstances, our author proceeds to estimate the advantage derived from the operation, and is obliged to confess that in some instances the ligature has not diminished the tumour. Hence it would appear that this operation of tying the superior thyroid artery, which can rarely be absolutely necessary, has sometimes destroyed the patient in a few days, and sometimes entirely failed in its object: and these discouraging results have occurred several times in a very small number of cases: we shall therefore pass over the rest of Dr. Beck's observations on the operation for *Struma aneurysmatica*, and proceed to analyse his remarks on another variety of bronchocele.

After observing that the most ordinary species is the *Struma lymphatica*, and quoting Langenbeck and others, who found these tumours filled with fluid, Dr. Beck says that he has paid particular attention to this subject; and, having frequently found tumours of this kind, both in living patients and in post-mortem examinations, he thinks it necessary to make another subdivision, under the name of *Struma cystica.*

Our author describes several anatomical preparations of this morbid structure, which were lying before him. The interior of the cyst was generally smooth, and like a serous membrane, *(Struma cystica membranacea,)* but in two cases it had become bony, *(Struma cystica cum ossificatione cystæ partiali aut universali.)*

In the six following cases Dr. Beck removed the cyst with the knife.

Case I. R. Z., of Freiburg, a boy twelve years old, of a lymphatic constitution, but without any marks of scrofula, was admitted into the clinical ward of surgery in 1826. In the front part of the neck there was a swelling, which was particularly developed on the right side, pushing the trachea and the thyroid cartilage to the left. The tumour protruded anteriorly and downwards, and pushed the Sternocleido-mastoideus outwards. It was tense and elastic, and a fluctuation could be perceived on pressure, though obscured by the tenseness of the tumour. Its edges were not sharply defined, but, on the contrary, depressed, and its limits could not be clearly distinguished. The skin was unaltered in colour and quality; the veins of the neck were enlarged, and the pulse of the carotid (but not of the superior thyroid) was stronger than natural. This tumour, which had existed for a year, and had not arisen from any apparent external influence, soon reached a considerable size. Its growth never stopped entirely, but for some months its increase had been inconsiderable. The patient complained of confusion and pain in his head, giddiness, and dyspnœa, which was worse at night. His voice had become somewhat fainter. Several physicians had prescribed the usual remedies employed against goître, but without effect.

The operation was performed on the 23d of November, in the presence of privy-councillor Ecker, and the students visiting the clinical ward. The patient was placed on a low chair, with his head fixed by an assistant, who stood behind him. The skin being drawn up, and held by an assistant, was divided by one stroke of the knife, forming an incision two inches and a half long. By the second incision the cellular substance and the platysma myoides were cut through, and the thyroid gland laid bare. Several laminæ of the gland, and probably also parts of the sternothyroideus were now removed, so that the cyst came nearer the surface, and the fluctuation could be plainly distinguished. The contained fluid was pushed forwards; and the point of the knife was plunged in at the most prominent part, until the opening of the cyst had been placed beyond a doubt by the cessation of all resistance, and by the fluid forcibly making its way out by the side of the blade. The incision was now enlarged to two inches in length, by means of scissors on a hollow sound. The fluid, which was somewhat turbid and glutinous, rushed out with great force, as it does when the incision is completed in the operation for hydrocele. The circumference of the part was considerably lessened immediately after the evacuation of the fluid. A long slip of linen, two inches broad, was inserted into the cavity, so as to fill it up, but with its extremity hanging out; the wound was then covered with adhesive plaster, and cold poultices were placed over the spot. The sac was perfectly smooth in its inner surface.

Soon after the operation (the hemorrhage during which was trifling,) the patient complained of pain, accompanied with a sense of tension: it was felt on the outside of the neck, and made swallowing difficult. Five hours after the operation, vomiting occurred, which was repeated, after which the patient fell into a refreshing sleep. The following day there was slight fever, and the local uneasiness continued. A sanguineo-serous secretion came out from the wound, but the dressings were not renewed. The treatment was antiphlogistic, but without abstraction of blood. On the third day, when suppuration had begun, the dressings were renewed, after the cavity had been cleaned out with an injection composed of honey and water.

"On the 28th of November, as the suppuration and local reaction seemed to be but slight, pledgets of lint dipped in Ung. de Styrace, were inserted into the cavity. This was the daily dressing, and after the linen had been taken out, infusion of chamomile, with rose honey, was employed as an injection. The treatment was continued in this manner until the 15th of December. In order to effect the destruction of the sac, and cause a more lively reaction in the surrounding parts, it was thought necessary to dip the pledgets into Ung. de Styrace mixed with red oxide of mercury. The sac came away piecemeal, the inner surface of the cavity granulated readily, and the pus was healthy. The cavity continued to diminish.

On the 15th of January superficial union had taken place, and cicatrization was promoted by touching the spot with lunar caustic. On the 20th cicatrization was completed; so that, by the end of the month of January, the patient was perfectly freed from this burdensome disease. The neck had recovered its due circumference, and the difficulty of respiration no longer existed. I can add, says Dr. Beck, that the consequences of the operation were permanent, as I frequently see the patient. A scar which remains at the anterior and lateral part of the neck, (but by no means a deforming one,) reminds one of the disease which existed, and of the operation by which it was removed.

Having given this case at great length, we must necessarily content ourselves with a brief abstract of the remaining ones narrated by Dr. Beck.

CASE II. Mr. E., of L., a young and active tradesman, applied to Dr. Beck, in the year 1827, for medical advice, on account of a *struma* which covered the right anterior part of the neck. The patient was hoarse, and not only suffered from dyspnœa, but from tinnitus aurium, epistaxis, and vertigo; his eyes too were affected with the appearance of flashing light: all these symptoms clearly showing the congestion of blood in the head. Dr. Beck proposed the operation, but the patient refused to consent to it. Some parts of the tumour were hard and unyielding, and, by striking it, a vibration could be perceived in several points. After trying a variety of treatment under different physicians for more than a year, the patient returned to Dr. Beck, but desired that, before the operation was performed, a consultation should be held on his case. This was accordingly done; and privy-councillor Ecker and professor Schwörer agreed with Dr. Beck in his opinion that this was a *Struma cystica*, combined with ossification, and only to be relieved by the knife. The operation was successfully performed, yet the results were not quite so satisfactory as in the former case; for, though the patient was able to return home in a few months, yet it was necessary, from the continued secretion, to keep the wound open; and two years afterwards it was found requisite to make a counter-opening behind the sternocleido-mastoideus, to give a freer passage to the laminæ of bone.

Dr. Beck then mentions a case of a young lady who was a remarkably fine singer, but, having lost her powers of song by a *Struma cystica*, regained them by the operation.

CASE III. E. K., a young lady of delicate constitution, first perceived, in November 1830, a slight goître on the left side of the neck. In December she suffered from a violent angina (cynanche tonsillaris), and the tumor after this increased so rapidly, that in March it was as big as a hen's egg. She took burnt sponge, and used frictions with iodic and mercurial ointment, but to no purpose. At one time an attempt was made to diminish the tumour by the simultaneous application of friction of pure iodine and the galvanic pile; half a grain of pure iodine, rubbed down with a little fat, being placed on one side of the goître, and the negative pole on the other. The operation was performed February 16th, 1832, and the result was a perfect cure. In this case also fragments of bone came away from the sac.

CASE IV. M. R., a strong and healthy girl, was operated on, October 20, 1831: the cure was perfect.

CASE V. J. S., aged twenty-two, entered the clinical ward of surgery, August 2d, 1831. The disease had existed for several years, and the best remedies had been employed for more than a year without success; further delay was therefore useless. It was thought expedient to operate on the day of his admission. Considerable hemorrhage occurred during the operation, making it necessary to take up an artery; but every thing went on so favourably, that he was discharged cured on the 3d of September.

CASE VI. E. V., aged twenty-four, a girl belonging to the middle ranks, entered the clinical ward on the 12th of June, 1833, suffering from a *Struma cystica*, combined with a *Struma lymphatica.* The operation was performed on the 14th. After opening the cyst, a portion of its parietes was cut off with the scissors; this was followed by considerable hemorrhage, and it was clear that part of the gland had been cut away also.* An artery which ran along the upper part of the wound was immediately tied. The hemorrhage lessened, but did not cease; and, as the cavity of the bronchocele continually filled with arterial blood, though not a single bleeding vessel could be found, it was plugged with balls of lint, sprinkled with Pulv. colophonii and gr. i. mimos; the wound was covered with lint sprinkled with the same powder; ice was applied, &c. The case went on quite favourably, and the patient left the

* The distress which a goître occasions to the patient is often caused only by a small portion of it. If this part is removed by the knife, the inconvenience ceases ; and, by the judicious application of a ligature to the portion about to be removed, all danger of hemorrhage is avoided. This is not a mere theory, but has been twice practically verified by a surgeon of our acquaintance.—EDITOR.

hospital on the 30th July. A fortnight after going out, perfect cicatrization had taken place, and the hardness at the lower part of the neck, which remained after the operation, yielded almost entirely to the use of an ointment containing hydriodate of potash, which had been employed without benefit before the operation.

After some observations on the diagnosis of the *Struma cystica* from the *Struma lymphatica*, and other similar affections, Dr. Beck remarks that Meckel mentions the occurrence of fungus hæmatodes of the thyroid gland, and then gives a case in which he believes this disease to have existed, though he had no opportunity of verifying his opinion by a post-mortem examination. The patient, a man of fifty, who had often suffered from pains in his limbs, was cachectic and feverish. The tumour was large, and covered with irregular prominences, one of which had opened two months before, and blood and a blackish brown fluid issued forth. A fungus soon made its appearance through this opening, which was nearly round, and its edges covered with a thin membrane. The patient returned to his place of residence, and Dr. Beck was informed of his death, which took place six weeks afterwards, by the physician to whom he had recommended him.

Dr. Beck disapproves of the seton as a means of curing bronchocele, and gives a case in which he employed it ten years ago, and its use was followed by fatal tetanus.

In the appendix our author gives another interesting case. The patient, a countryman, aged twenty-nine, entered the clinical ward, December 8th, 1832, suffering from a bronchocele of enormous size. The tumour on the left side ascended as high as the ear, and descended under the clavicle into the cavity of the thorax. An operation was quite out of the question, and the unhappy patient died suddenly of suffocation, on the tenth day after his admission.

The post-mortem appearances are given by our author with German fidelity and minuteness, (p. 71—76,) but are far too long to quote here. The chief point in the *struma* was, that great part of it consisted of a substance like brain that is nearly putrid. The principal morbid appearances in other parts of the body were, the scar of an old ulcer in the lungs, and a double pelvis (with a double ureter) in the right kidney.

Had we translated all that is worth reading in this essay, we should have translated the whole. We now take leave of Dr. Beck, with the highest respect for his industry, and admiration of his skill.

